# Subungal squamous cell carcinoma of the thumb – Implications for patient setup and radiotherapy planning

**DOI:** 10.1016/j.tipsro.2020.11.001

**Published:** 2020-12-03

**Authors:** Niall O'Dwyer, Karen Olden, Adrian J. Cubbin, Paul Hill, Aileen Flavin, Kathy Rock

**Affiliations:** Department of Radiation Oncology, Cork University Hospital, Cork, Ireland

**Keywords:** Subungal squamous cell carcinoma, Patient setup, Skin cancer, Radiotherapy

## Abstract

Subungal squamous cell carcinoma (SSCC) is a rare malignancy of the nail bed. We present the case of a woman diagnosed with a SSCC of the right thumb. Initial management consisted of surgically excision followed by adjuvant radiotherapy due to a positive bone margin. We describe the patient set-up, immobilisation technique, target volume delineation, radiotherapy planning, fractionation schedule, image guidance and patient outcomes.

## Highlights

• Radiotherapy is a suitable option for adjuvant treatment of unresectable SSCC.
• The radiotherapy dose was 55 Gy in 20 fractions.
• Case report details include patient setup and radiotherapy treatment plan.
• The patient remains disease free at 18-months post treatment.

## Introduction

Tumours located in the nail bed include squamous cell carcinoma (SCC), Bowen’s disease, melanoma and keratoacanthoma. The most common nail bed tumour is subungal squamous cell carcinoma SSCC which affects the fingers more commonly than the toes. The tumour is most commonly reported in middle-aged Caucasian men and risk factors include trauma, radiation exposure, chronic inflammation, smoking and more recently with Human Papilloma Virus type 16 and 18 [1], [2]. Diagnosis is often delayed in part due to a paucity of clinical awareness and also as the condition is mimicked by other conditions such as psoriasis or nail dystrophy. Suspicion of malignancy usually arises in the setting of multiple failed antibiotic and antifungal therapies with some cases reporting a delay of up to 4 years before a correct diagnosis is made [2]. Investigations include biopsy (punch/excisional/tangential), MRI and/or ultrasound to assess exact anatomical location and status of regional lymph nodes.

Due to the rarity of published data our objective is to report the case of a patient with SSCC that underwent primary surgical excision with a positive bone margin. We describe adjuvant radiotherapy details including patient set-up, immobilisation technique, planning and fractionation schedule along with patient outcomes.

## Ethics

Ethical approval was granted by the Cork clinical research ethics committee.

## Case

A 72-year-old right-handed female presented with a 18 month history of a non-healing area in the nail bed of the right thumb. Numerous antifungal and antibiotic therapies were prescribed without success. The patient was a non-smoker, not diabetic with no history of previous immunosuppressant medications. Physical exam revealed a dystrophic nail of the right thumb, with underlying subungal keratosis. All other nails of both hands and feet were normal. Assessment of the arm and axilla revealed no lymphadenopathy.

## Initial treatment

**Fig. 1:**
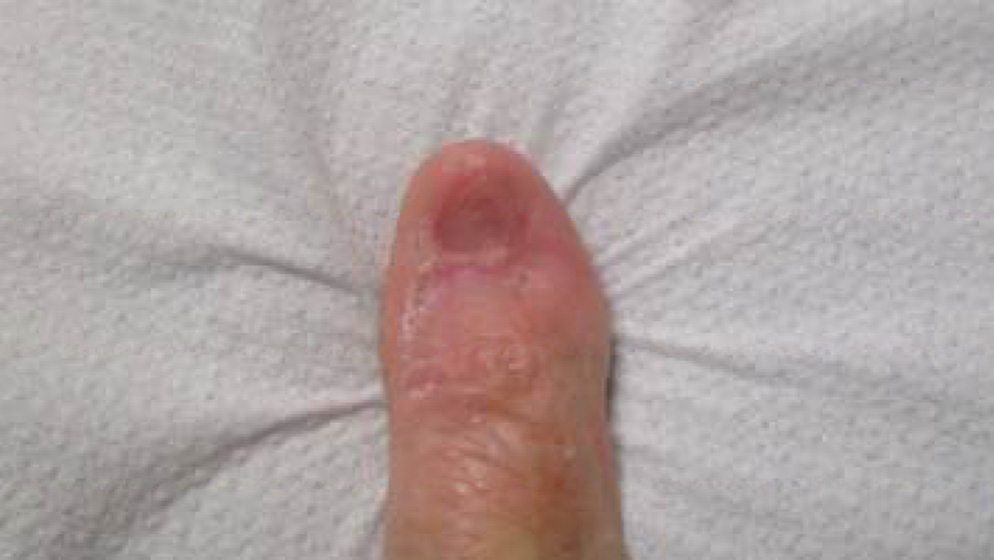
Post-excision pre-radiotherapy image of right thumb.

Initial biopsy revealed a moderately differentiated invasive squamous cell carcinoma. A subsequent extended wide local excision under local anaesthesia identified a 13 mm invasive SCC with 2.5 mm depth of invasion, no perineural or lymphovascular invasion. The bone margin was positive and the patient was referred for adjuvant radiotherapy. Mohs micrographic surgery a specialised surgical technique was not readily accessible at the time of this patient’s operation Please refer to Fig. 1 for an image of the right thumb post-excision pre-radiotherapy. HPV status was not assessed in this case as it would not affect the radiotherapy dose or setup. Prior to initiation of adjuvant radiotherapy MRI of the thumb was completed to excluded local disease and bone involvement (CT imaging could also have been considered). Ultrasound of right epitrochlear and axillary region did not reveal any local or regional spread thus the risk of distant metastatic spread was considered to be low and PET CT was excluded.

## Patient set-up and immobilisation technique

**Fig. 2:**
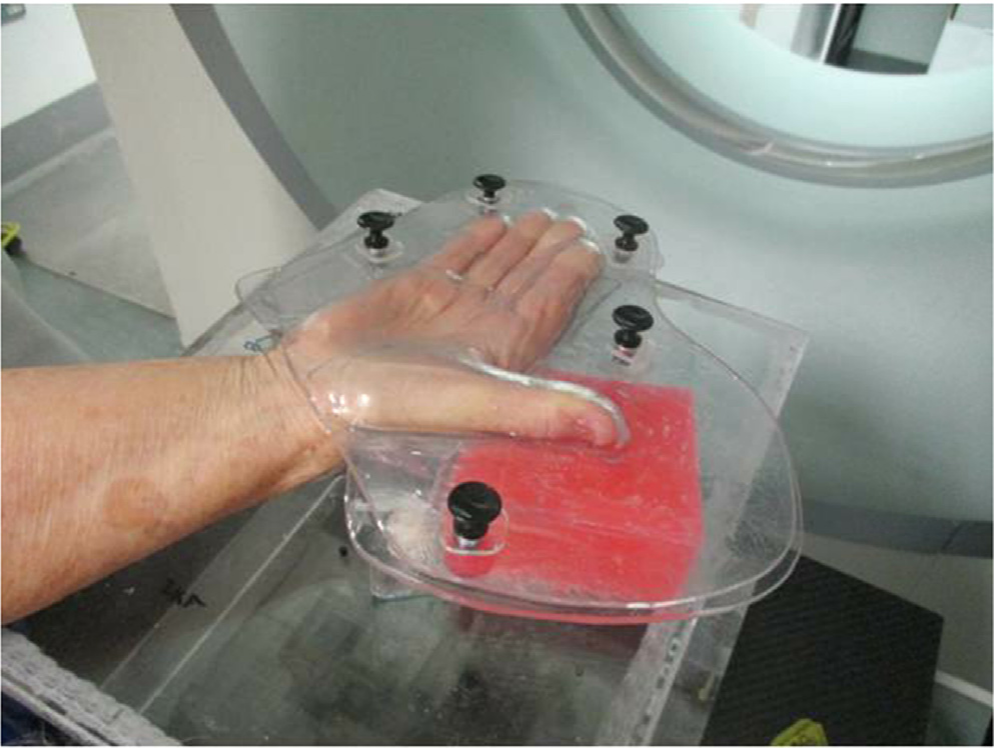
Back of hand is placed in bottom half of open immobilisation device, then the top half is positioned and clipped to bottom half using black clip-fasteners, thereby holding the hand securely between. The bottom red wax block can be left permanently in place. (For interpretation of the references to colour in this figure legend, the reader is referred to the web version of this article.)

**Fig. 3:**
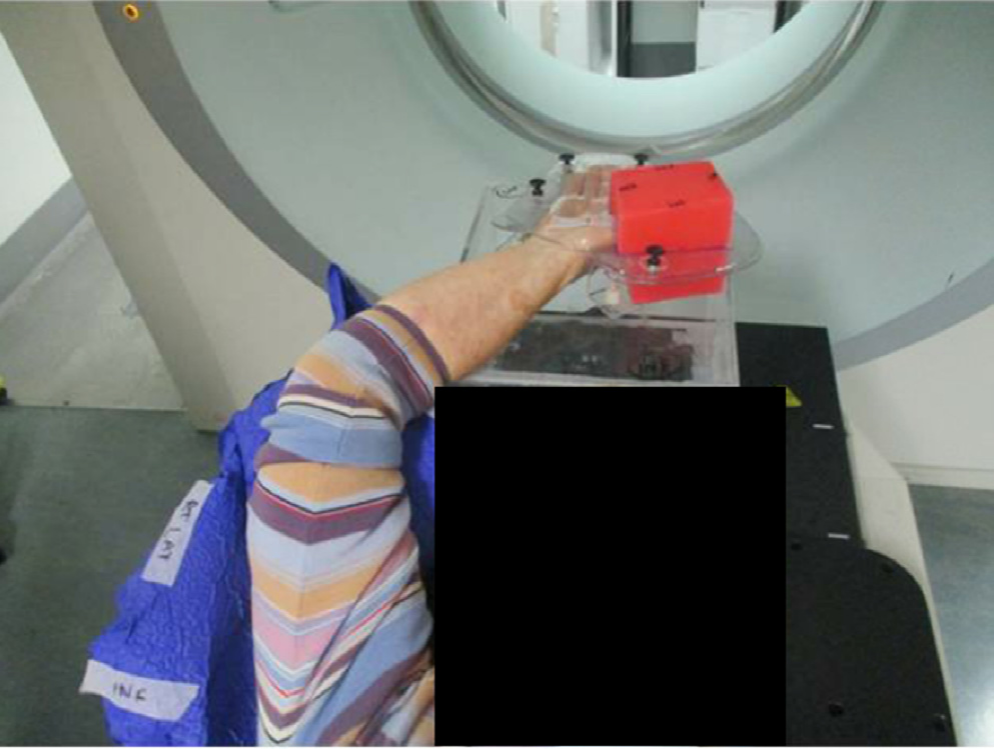
Top red wax block is positioned over thumb ready for treatment. A blue vac-lok cushion is used to support the elbow and upper arm. The device was secured to a raised Perspex block as patient was unable to rotate shoulder sufficiently to allow hand to rest directly on treatment couchtop. (For interpretation of the references to colour in this figure legend, the reader is referred to the web version of this article.)

The patient consented to adjuvant radiotherapy to reduce the chance of a local recurrence and as an alternative to further surgery which would likely have been an amputation. The patient was positioned in the supine position with the right arm flexed at 180 degrees, the elbow in 30 degrees of flexion and the wrist pronated so the palm was facing upwards. The hand and thumb were immobilised in a Perspex cube block attached to an Civco S-board using the board’s locating pins. The hand and thumb were immobilised using a two-part PETG (plastic polyethylene terephthalate glycol) device clipped together with detachable clip fasteners. This in turn rested on an in-house fabricated Perspex box attached to a CIVCO S-board using the board’s locating pins. The plastic two-part mould was made by taking an impression of both sides of the hand using dental impression alginate and plaster of Paris bandages. From this, positive plaster moulds were constructed which in turn were vacuum-formed to produce the plastic immobilisation device. Fig. 2, Fig. 3 illustrate the two removable blocks that were added top and bottom so that the thumb was tightly encased in was thus providing a homogenous radiotherapy dose.

If the patient is unable to raise their hand above their head it is possible to replicate the above set up (with or without the Perspex box) with the hand pronated and the arm in partial abduction away from the body. Due to the constraints of the CT bore the hand should not extend more than 30 mm beyond the lateral edge of the patient bed. This may require the patient’s body to be positioned as far laterally away from the treated hand as the width of the bed will allow. To ensure greater patient comfort and safety and to immobilise the arm securely a large vac-lok bag should be positioned under the patient’s torso and arm. As the CIVCO board cannot be utilised in this configuration it will also be necessary to construct a large Perspex baseplate (10 mm thickness) onto which the immobilisation device can be securely attached. This should be large enough to extend under the patient (and vac-lok bag) to cover the full width of the bed. It should also be constructed with locating blocks attached so that the baseplate can be secured to and be reproducible with the vak-lok bag. Whilst this option is feasible it does place constraints on treatment planning options as it does not allow full 360 degree treatment access.

## Radiotherapy planning

**Fig. 4:**
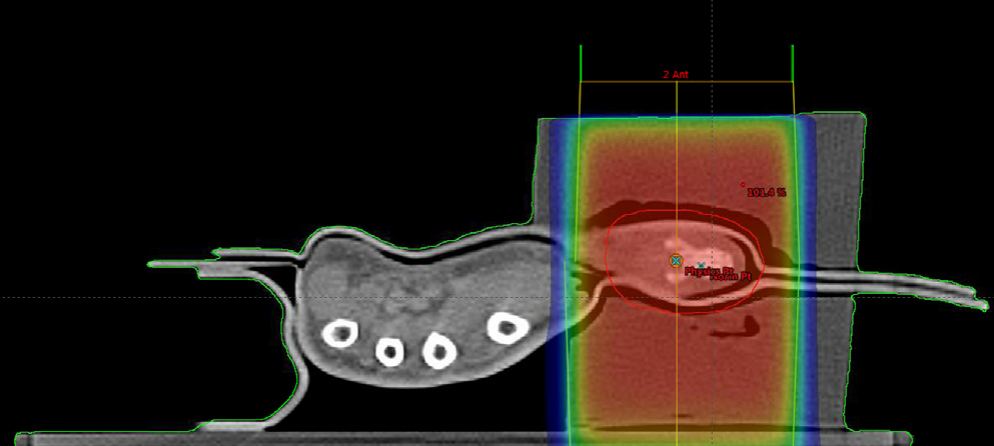
Axial plane of the planning CT showing the patients hand encased in the wax block and the dose fully covering the PTV (red outline). (For interpretation of the references to colour in this figure legend, the reader is referred to the web version of this article.)

Clinical target volume (CTV) was delineated with reference to the operative, pathological reports and clinical examination. The final CTV was 10 mm around the surgical bed curtailed at air. This was expanded by 5 mm circumferentially to create the planning target volume (PTV). The Eclipse (Varian Medical Systems) Analytical Anisotropic Algorithm (AAA) version 13.6 was used to create a 3-D conformal external beam radiotherapy with 6-MV photons. An AP/PA beam configuration was employed to fully cover the PTV while avoiding the non-involved portion of the hand. Fig. 4 illustrates the dose distribution in the axial planes. It can be seen how the addition of the wax blocks provide full scatter conditions to ensure adequate coverage of the PTV. The minimum dose to the PTV was 95% and the maximum dose 101%. A hypofractionated schedule of 55 Gy in 20 fractions (2.75 Gy per fraction) was chosen as the volume was small and the patient was travelling a distance to the radiotherapy department. On treatment images were taken days 1–3 and then 4 days later and weekly thereafter. Immobilisation was extremely satisfactory with negligible movement (<1mm on average).

## Patient outcome

MRI six months post completion of treatment showed no evidence of local recurrence. Clinical follow up at 6, 12 and 18 months showed no evidence of disease recurrence. The patient retained full range of motion of the joint with no late radiotherapy toxicity affecting function or ability to complete activities of daily living.

## Discussion

This case highlights the complexities around treatment of SSSC. It is a rare disease and thus there is a paucity of information on the condition and treatment algorithms. A small number of case reports have demonstrated radiation therapy as a suitable option for salvage of unresectable SSCC as an alternative to amputation [3]. As digits are not a common treatment site in radiotherapy departments this case highlights radiotherapy challenges including accurate target volume delineation, CTV-PTV expansion, elective nodal basin treatment, radiotherapy technique, patient set-up and possible impact of air gaps during planning along with choice of dose/fractionation schedules.

To aid in delineation of CTV we took into account the available tumour and patient information. A 10 mm expansion from the surgical bed was used to account for possible subclinical spread. As the patient set up and immobilisation technique were satisfactory we did not anticipate a large variation in daily set-up thus a 5 mm CTV-PTV margin was employed. The decisions on CTV and CTV to PTV margins were similar to previously published data [4] for non-melanoma skin cancer. Target volume delineation, CTV-PTV expansion and plan parameters were reviewed at our multi-disciplinary institutional peer-review quality assurance rounds.

When considering the option of treating the regional lymph nodes we considered the favourable tumour factors in this case including lack of perineural or lymphovascular invasion. We deemed the potential increased morbidity of elective regional lymph node radiation (risk of lymphedema) to outweigh the possible benefits and no elective radiation of the regional lymph nodes was undertaken in this case. Two years later in 2020 the American Society for Radiation Oncology (ASTRO) published clinical practice guidelines [5] which suggests that there is limited data to support use of elective lymph node basin radiotherapy even in patients at high risk for recurrence.

A number of radiotherapy techniques could be considered in this case including brachytherapy, electron planning or external beam radiotherapy with volumetric-modulated arc therapy (VMAT). A 3-D conformal photon plan was chosen as it achieved adequate coverage, was widely available and supporting case studies[6] have shown that photon plans can have dosimetric advantages over electron plans. Radiotherapy can be used as a safe alternative primary treatment for patients with finger SSCCs who chose not to undergo surgery [7]. A 2007 study from the Netherlands Cancer institute showed 92% permanent local control in 12 patients who underwent primary radiotherapy for finger SSCC [8]. No regional or distant failure was observed during follow-up, and the single patient that was found to have a recurrence underwent an amputation of the digit.

The dose calculation algorithm used was the Eclipse (Varian Medical Systems) Analytical Anisotropic Algorithm (AAA) version 13.6. AAA was developed to improve the dose calculation accuracy, especially in heterogenous media [9]. There are gaps between the bolus and PETG of up to 5 mm caused by the wax cooling. The dose in the air gap is not considered important, but rather the dose in the tissue distal to the air gap. The AAA is a type-B algorithm which models the lateral electron transport. It has been shown that models of this type perform similarly to the Monte-Carlo methods in determining the dose distal to a tissue inhomogeneity so it would be expected that any additional dose would be small. Measurements also indicate that for the field sizes in this case the dose distal to a gap is largely unaffected for gaps of this size

The Royal College of Radiologists (RCR) published guidelines on radiotherapy dose fractionation for squamous cell skin cancer [10] proposing a wide variety of acceptable dose and fractionation options. There are no randomised control trials examining dose-fractionation, all studies examined were Oxford CEBM Grade C evidence. In this case taking into account patient factors including proximity to the hospital, site of treatment and field size a hypofractionated schedule of 55 Gy in 20 fractions (2.75 Gy per fraction) was chosen in keeping with RCR supported fractionation schedules.

Weekly clinical review was undertaken during treatment and acute toxicities (Common Terminology Criteria for Adverse Events version 4 [11]) recorded. The maximum acute toxicity recorded was grade 1 radiation dermatitis during the final week of treatment which was managed successfully with topical emollient. The patient retained good range of motion and function of the thumb throughout treatment and at each assessment up to 18 months post- treatment. Longer follow up will be required to further assess for late radiotherapy toxicities.

## Conclusion

SSCC represent a real therapeutic challenge. Adjuvant radiotherapy is an alternative to limb amputation. This patient was comfortably immobilised during treatment, did not suffer any significant toxicity and remains disease free at 18 months with full joint mobility and function.

## Declaration of Competing Interest

The authors declare that they have no known competing financial interests or personal relationships that could have appeared to influence the work reported in this paper.
